# Hybrid mosquitoes? Evidence from rural Tanzania on how local communities conceptualize and respond to modified mosquitoes as a tool for malaria control

**DOI:** 10.1186/s12936-021-03663-9

**Published:** 2021-03-06

**Authors:** Marceline F. Finda, Fredros O. Okumu, Elihaika Minja, Rukiyah Njalambaha, Winfrida Mponzi, Brian B. Tarimo, Prosper Chaki, Javier Lezaun, Ann H. Kelly, Nicola Christofides

**Affiliations:** 1 Environmental Health and Ecological Science Department, Ifakara Health Institute, P. O. Box 53, Ifakara, Tanzania; 2 School of Public Health, Faculty of Health Sciences, University of the Witwatersrand, 1 Smuts Avenue, 2000 Braamofontein, South Africa; 3 Institute of Biodiversity, Animal Health and Comparative Medicine, University of Glasgow, Glasgow, G12 8QQ UK; 4 School of Life Science and Bioengineering, The Nelson Mandela African Institution of Science and Technology, P. O. Box 447, Arusha, Tanzania; 5 Institute for Science, Innovation and Society, School of Anthropology and Museum Ethnography, University of Oxford, Oxford, UK; 6 Department of Global Health and Social Medicine, King’s College London, London, UK

**Keywords:** Malaria elimination, Genetically-modified mosquitoes, Gene drives, Public perceptions, Community engagement

## Abstract

**Background:**

Different forms of mosquito modifications are being considered as potential high-impact and low-cost tools for future malaria control in Africa. Although still under evaluation, the eventual success of these technologies will require high-level public acceptance. Understanding prevailing community perceptions of mosquito modification is, therefore, crucial for effective design and implementation of these interventions. This study investigated community perceptions regarding genetically-modified mosquitoes (GMMs) and their potential for malaria control in Tanzanian villages where no research or campaign for such technologies has yet been undertaken.

**Methods:**

A mixed-methods design was used, involving: (i) focus group discussions (FGD) with community leaders to get insights on how they frame and would respond to GMMs, and (ii) structured questionnaires administered to 490 community members to assess awareness, perceptions and support for GMMs for malaria control. Descriptive statistics were used to summarize the findings and thematic content analysis was used to identify key concepts and interpret the findings.

**Results:**

Nearly all survey respondents were unaware of mosquito modification technologies for malaria control (94.3%), and reported no knowledge of their specific characteristics (97.3%). However, community leaders participating in FGDs offered a set of distinctive interpretive frames to conceptualize interventions relying on GMMs for malaria control. The participants commonly referenced their experiences of cross-breeding for selecting preferred traits in domestic plants and animals. Preferred GMMs attributes included the expected reductions in insecticide use and human labour. Population suppression approaches, requiring as few releases as possible, were favoured. Common concerns included whether the GMMs would look or behave differently than wild mosquitoes, and how the technology would be integrated into current malaria control policies. The participants emphasised the importance and the challenge of educating and engaging communities during the technology development.

**Conclusions:**

Understanding how communities perceive and interpret novel technologies is crucial to the design and effective implementation of new vector control programmes. This study offers vital clues on how communities with no prior experience of modified mosquitoes might conceptualize or respond to such technologies when deployed in the context of malaria control programmes. Drawing upon existing interpretive frames and locally-resonant analogies when deploying such technologies may provide a basis for more durable public support in the future.

## Background

Malaria is thought to have killed between 150 million and 300 million people worldwide during the twentieth century [1]. Although the situation has improved in the last two decades, malaria remains one of the leading causes of death and ill-health globally [2]. In 2019 more than 200 million people were diagnosed with malaria and nearly half a million died, more than 90% of whom lived in sub-Saharan Africa (SSA) [2]. Interventions such as insecticide-treated nets (ITN) and indoor residual spraying (IRS), combined with improved diagnosis and treatment account for most of the reductions in malaria burden [3]. Yet these interventions appear to have reached the limit of their efficacy in many regions [4–7]. Achieving further gains and not losing ground in the fight against the disease will require the development of novel and complementary interventions [8–10].

Mosquito modification technologies have garnered a great deal of public interest, particularly in SSA, where their impact is expected to be highest as a tool for malaria control and elimination [9, 11–13]. While experiments with some of these technologies, particularly the Sterile Insect Technique (SIT), go back several decades [14], significant progress has been made recently in the development and evaluation of novel approaches [15, 16] such as the Release of Insects carrying a Dominant Lethal genes (RIDL) [17], gene-drive technologies [15, 18–21], or the release of mosquitoes infected with *Wolbachia* bacteria and other endosymbionts [22–24].

These technologies are at different stages of development, and face specific questions from the perspective of communities considering their introduction. One important distinction is between interventions aiming to eliminate the relevant mosquito species (population suppression), and those intended to permanently introduce a novel mosquito strain that will block or interfere with pathogen transmission (population replacement) [15]. These differences suggest the need for distinct communication strategies, and imply a very different set of expectations on the coexistence between modified mosquitoes and the communities hosting the intervention [25].

Given the promise attributed to these technologies, their purported high-impact, and the numerous uncertainties that still surround their future deployment, extensive stakeholder engagement is essential in order to identify potential obstacles and concerns in malaria-endemic regions [15, 26, 27]. Opposition to the release of genetically modified mosquitoes in south-east Asia and the Americas [28–30], and evidence of concerns among stakeholders in Mali [31], Nigeria [32] and Tanzania [33] suggest the importance of proceeding with caution [26, 27]. Robust social scientific research into how these novel technologies are perceived in areas where they might be deployed is a prerequisite for an effective public engagement strategy [34].

This study investigated community awareness and perceptions of genetically-modified mosquitoes (GMMs) and their potential for malaria control in south-eastern Tanzanian villages where no research or campaign for the introduction of such technologies is currently underway. To examine how a typical malaria-endemic community might respond to the introduction of GMMs technologies, the study explored the different conceptual frameworks and analogies that communities use to make sense of modified mosquitoes as a tool for malaria control.

## Methods

**Fig. 1:**
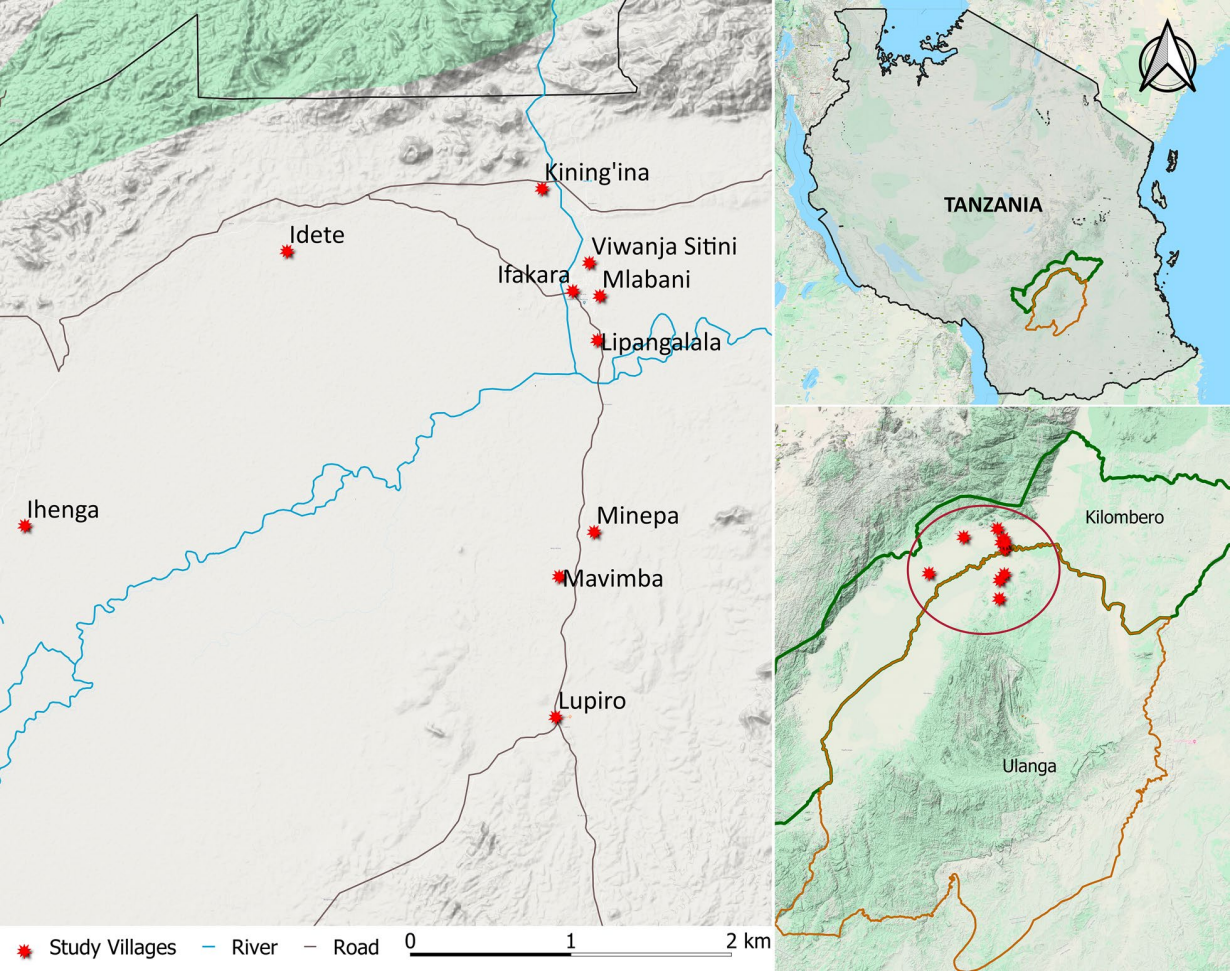
Map of the districts and villages where the study was conducted. Map prepared by Najat Kahamba

This study was part of a larger public engagement process aiming to understand and improve public awareness and community evaluation of alternative interventions for malaria control and elimination. This particular study was carried out in ten randomly selected villages in two districts in south-eastern Tanzania between May and December 2019 (Fig. 1). Detailed description of the villages is provided by Finda et al. [5, 35], Kaindoa et al. [36] and Mmbando et al. [37]. Although this area has previously hosted numerous malaria research projects, there had not been any research on modified mosquitoes of any kind up to that point. Previous studies in the area have demonstrated high levels of knowledge about mechanisms and patterns of malaria transmission [5, 38, 39].

### Study design and data collection

An exploratory sequential mixed-methods approach [40] was used. Focus group discussions (FGDs) were held with community leaders from each of the ten selected villages to explore in detail their perceptions of mosquito modification. Community leaders are governmental officials elected by the community members every 2 years, and represent their respective communities in several district- and regional-level meetings. They do not belong to any political party; their responsibilities include resolving conflicts, authorising property sales, and monitoring migration in and out of their communities. Two community leaders, one male and the other female, were selected per village. Two separate FGD sessions were conducted, one with female and another one with male leaders, and were facilitated by MFF and a research assistant in Swahili language. The sessions were held in May 2019. Each session took around 2 h. The discussions were structured to elicit vernacular modes of reasoning about mosquito modification and the prospect of releasing altered mosquitoes to combat malaria. Specific attention was paid by the moderator to the analogies and examples that participants used to characterize GMMs.

Due to the low levels of awareness of mosquito modification technologies, FGD participants were provided with a brief PowerPoint presentation on mosquito modification to prompt and facilitate informed discussions. The presentation covered different approaches (i.e., sterile insect technique, male RIDL mosquitoes, and gene drive technology). The presentations also included basic information on how the mosquitoes are modified and released, and the current stage of development of each approach. These materials were designed to avoid any value judgment on the potential of any particular approach, so as to preempt, to the extent possible, any interpretive bias among participants. The discussions were guided to elicit participants’ views on each of the mosquito modification technologies, including any perceived risks and benefits, and on the factors that might determine acceptance by the local community.

Preliminary findings from the FGDs were used to develop a structured questionnaire to measure prior awareness, knowledge and perceptions of mosquito modification technologies for malaria control among the broader community. The survey was administered to community members in the ten selected villages. According to data from the Ifakara Health and Demographic Survey System [41], the selected villages encompass a total of 11,000 households. Assuming a response rate of 80% and 95% confidence interval, it was estimated that a sample size of 463 household representatives would be needed. This number was rounded to 500 representatives to account for lack of consent. The 500 households were equally divided between the villages; 50 households were randomly selected in each of the ten villages, and were visited by the study team accompanied by community leaders. One consenting adult in each household was interviewed. The survey was carried out between November and December 2019, and was administered using Kobotoolbox™ software [42] on electronic tablets. The study team asked the respondents questions and recorded their answers on the tablets.

### Data processing and analysis

The proceedings of the FGDs were transcribed and analysed by MFF, EM, RN and WM. Verbatim transcriptions of the FGDs were translated from Swahili to English, and imported into NVIVO 12 Plus software [43] for coding. Both deductive and inductive coding were used. The FGD guide was used to develop deductive codes, but since the technologies under discussion were new to the participants most of the codes were generated inductively after extensive reviews and coding of the transcripts. Recurrent themes were extracted from the emergent patterns. Direct quotes from FGD participants are used below to illustrate some of the key themes.

R statistical software version 4.0.0 [44] was used to analyse the socio-demographic characteristics of the survey respondents, and to summarise their knowledge and awareness of GMMs. Since a vast majority of respondents lacked knowledge and awareness regarding the technology, no further analyses were necessary. Instead, lay presentations about the technologies were provided to prime further discussions in the FGDs.

## Results

### Characteristics of study respondents

A total of 506 people participated in this study; 16 community leaders who took part in the two FGD sessions, and 490 community members who responded to the survey. Three of the FGD participants had secondary school education (12 years of formal education), and the rest had primary school education (7 years of formal education).

A detailed description of the survey respondents is provided in Table 1. The mean age was 42.5 years (range: 18–88), and were about equally divided between men and women. A majority of the respondents were married, had primary school education, and reported farming as their main income generating activity (Table 1). The reported average monthly household income was 132,155 Tanzanian shillings (~ 60 USD).

**Table 1:** Socio-demographic characteristics of the survey respondents

Characteristics	Category	n (%)
Age (in years)	18–35	186 (37.9%)
	36–55	207 (42.3%)
	56–88	97 (19.8%)
Marital status	Married	321 (65.5%)
	Not married	82 (16.7%)
	Divorced/separated	39 (8.0%)
	Widow/widower	48 (9.8%)
Highest educational level achieved	No formal education	43 (8.8%)
	Primary school	358 (73.0%)
	Secondary school	68 (13.9%)
	College/university	21 (4.3%)
Main income generating activities^a^	Farming	413 (84.3%)
	Entrepreneurship	174 (35.5%)
	Fishing	12 (2.4%)
	Animal husbandry	23 (4.7%)
	Formal employment	13 (2.7%)

^a^The totals add up to more than 100% because some participants chose to report more than one income generating activities

### Community awareness of malaria burden

Previous surveys in the study area have shown high levels of awareness among residents of these communities about malaria and its transmission by *Anopheles* mosquitoes [5, 45, 46]. In this study, two thirds of the respondents (65.1%, n = 319) believed that rural communities experienced higher burden of malaria, 63.9% (n = 313) believed that poor communities experienced a higher burden of malaria, and 61.3% believed that transmission occurred mostly outdoors. However, when asked about specific details, only 15.3% (n = 75) had a good estimate of current malaria prevalence in the country (as reported in the 2018 Malaria Indicator Survey report [47]). Half (51.6%, n = 253) of all respondents believed that the country was making good progress in malaria control. 59.6%, (n = 292) believed that it was possible to achieve elimination with the current interventions, but 86.1% (n = 422) of respondents indicated that alternative interventions would be necessary to accelerate elimination efforts.

### Community views on novel interventions for malaria control

All survey participants responded that any new technologies for malaria control should be effective, affordable, meet in-country regulations and community preferences, and be safe to people, animals and the environment. When asked about trusted sources of malaria-related information, health researchers and health care workers were ranked higher than government officials or politicians (Table 2).

**Table 2:** Community members’ levels of trust for sources of information on malaria control interventions (N = 490)

Variables	Highly trusted (%)	Somewhat trusted (%)	Somewhat distrusted (%)	Strongly distrusted (%)
Health researchers	91.2	7.6	0.4	0.8
Health care workers	91.2	8.2	0.4	0.2
Government officials	84.9	12.7	1.6	0.8
Politicians	55.3	26.1	9.0	9.6

### Awareness of mosquito modification technologies for malaria control

A vast majority of survey participants (94.3%, n = 462) reported no prior awareness of mosquito modification technologies for malaria control. For the 13 respondents who were aware, the primary sources of information were Ifakara Health Institute staff, and radio or television. Likewise, nearly all participants 97.3% (n = 477) reported no knowledge of how any of these technologies worked. When asked if they thought modified mosquitoes had ever been released in their communities, 83.5% (n = 409) said they did not know and 16.5% (n = 81) said they had not been released.

### Community leaders’ perceptions of mosquito modification

None of the community leaders who participated in the focus group discussions reported any prior knowledge of mosquito modification technology. They were able to discuss the subject at length and in detail, however, once they were provided with a brief presentation of issue. They often expressed a great deal of fascination over this approach to malaria control, preferring it over other malaria control interventions. Key attributes of the technology mentioned to justify this preference were the improvement of environmental safety (as a result of reducing the use of chemical insecticides), and the little effort the technology appeared to require from local residents (in contrast to other malaria control methods, such as larviciding or home improvements, deemed more labor intensive).

Although three distinct approaches of mosquito modification were presented to FGD participants, participants showed a clear preference for discussing gene drive technologies, and in particular the male-biased sex distorter gene drive that is currently being considered for deployment in several sub-Saharan countries at the moment [48]. Gene drive technology was preferred because it was seen to require fewer releases of modified mosquitoes compared to the other two, a fact that participants thought would help reduce community skepticism towards the intervention.

“*It is better if you do not release mosquitoes all the time. Even if people agree that you release mosquitoes, if you do it a lot they may start asking questions again, then you have to spend a lot of time convincing them. But I like this one that does not kill mosquitoes, but makes them have male babies. With this one you can do it just one time, then it is good.”* (Female).

As the above quote suggests, several participants were intrigued by the idea of eliminating mosquitoes by biasing the sex distribution of their offspring, rather than by killing them directly. This was in some cases considered a more humane way of eliminating the mosquitoes.

*“I really like the idea of making them have just male babies, because, you see, males do not bite, and without females they cannot have babies. This way even your consciousness is clean, you have not killed them directly, you have just manipulated them and they will eventually die off. This is a very good and very advance technology”* (Male).

### Framings and analogies used to describe mosquito modification

Although FGD participants were unfamiliar with mosquito modification, they immediately grasped its public health logic by reference to their knowledge of cross-breeding and hybridization. Several participants indicated that the best way to explain this technology to people in the community would be to describe it as a form of ‘*kupandikiza*’, a term that can be literally translated as transplantation but is commonly used to describe hybrid plants. The term was used, without any prompt from the facilitator, in both FGD sessions. Participants used the example of the hybrid maize seeds that they buy in agricultural shops, which have a relatively higher yield and can better withstand drought than local maize varieties. FGD participants also referred to the technology as *‘kubadilisha mbegu’*, the practice of ‘changing seeds.’ The term is generally used to describe the introduction of desirable traits in crop seeds and domestic animals through cross-breeding. Several participants mentioned for example that they often borrow or pay for the use of their neighbours’ male animals in order to get offspring with the desired traits.

*“I do it often with my chickens. I don’t have a strong rooster, but my neighbour has a very big one. So I ask my neighbour for her rooster to spend time with my chickens, then I can get its seeds. Everyone does that.”* (Female).

*“It is very common with pigs. Sometimes there is one person in the village who has a very big boar, so then, if you want to get its seeds you pay that person money so that the boar can mate with your sows. Sometimes you pay money or sometimes you pay him with a litter. But we do that so that we can have the seed for big pigs.”* (Male).

### Will the modified mosquitoes look and behave differently?

Participants expressed curiosity and concern over the appearance and behaviour of the modified mosquitoes. They wondered, for example, whether or not the mosquitoes would look the same as ‘local’ mosquitoes. Participants drew again an analogy with their experience of selectively-bred animals or hybrid maize, and concluded that the modified mosquitoes would necessarily look different.

*“Yes, they always look different. Even when we plant the hybrid maize, it does not look the same as our local maize, it has better yield, and you can tell just by looking that it is different kind of maize.”* (Female).

Village leaders were also keen to know whether modified mosquitoes would still bite people, and whether or not current mosquito control tools could or should be applied to them.

*I would like to know, if you want those traits to pass to their offspring, will we still need to kill these modified mosquitoes? Will they still bite people? If they bite, people will still want to kill them, and if they do, then it may not work.”* (Male).

### All mosquitoes are a nuisance; why not just eliminate all of them?

A majority of FGD participants suggested that technologies of mosquito modification should target all mosquitoes, and not just those transmitting malaria. This line of argument was particularly relevant for genetic modification approaches aimed at population replacement, and participants expressed the fear that modified mosquitoes, if they became a feature of the environment, would still be able to carry other pathogens. Additionally, participants stressed the fact that mosquitoes are always a nuisance, regardless of the species; their bites are itchy, painful and cause allergies, so it would be beneficial to just eliminate them altogether. Some participants drew a direct analogy with their experience of jiggers (*Tunga penetrans*) and lice, which were once prevalent in the region but have been eliminated altogether in their communities. They expected a similar sort of objective should be pursued in the case of mosquitoes.

*“We should just eliminate all mosquitoes, the way jiggers were eliminated. In the past there were so many jiggers; as kids we had to go to the hospital to get them removed from our feet. But then something was done and they all disappeared. These days you never hear about them, and the children these days do not even know what jiggers are. I would like that to be the case with mosquitoes, all of them. I would be happy if the future generations do not know anything about mosquitoes, maybe they should only see them in the pictures.”* (Male).

FGD participants drew a direct connection between the effectiveness of the intervention and a reduction in the overall density of mosquitoes. They argued that people would only have faith in the merits of the technology if they saw a substantial reduction in nuisance biting. They further noted that most people are unable to distinguish between malaria vector and non-vector mosquito species, and thus would fail to appreciate the impact of the intervention if it was limited to a single species.

*“But why would you want the other mosquitoes to remain? For me that is a challenge, that there will still be mosquitoes. People may think that it is not working. The other technologies kill mosquitoes, so then you will know that mosquitoes are not as many. But with this technology there will still be mosquitoes – even if they do not spread malaria, but people will not know that.”* (Female).

A few participants, however, did note that mosquitoes also have a place in the ecosystem, and thus supported the idea of eliminating only those responsible for malaria transmission. They pointed out that it would be impossible to eliminate all mosquitoes, because they had never been to or heard of a place where they are completely absent. They further expressed the view that it would be highly important to inform the community that not all mosquitoes would be eliminated, just the ones that spread malaria, so as to prevent mistrust of the technology.

*“I do not think there is a need to eliminate all the others if they are not transmitting anything. Remember, there are other birds and other insects that feed on mosquitoes, so it is no use to kill something that is harmless. You know, even in countries that do not have malaria there are still mosquitoes. I know this. So then it is okay to have mosquitoes that do not have malaria. You just need to teach people to differentiate malaria mosquitoes from other mosquitoes so that they know the difference.”* (Male).

### Importance of engaging and educating community members

All FGD participants stressed the importance of educating and engaging the community in the development of these technologies. They emphasized that this should be done not just once but repeatedly until their level of awareness and knowledge was such that they could participate in any decision to bring the technology into the community.

*“It is just very important to make sure that people are well aware of this technology. You have to educate them well. Tell people the benefits of this science, and the risks of continuing to have malaria mosquitoes. I think people should know what can happen if people agree to have these mosquitoes released, and what will happen if they do not. For example, you can talk to people maybe two or three times every month, and do it like that until it becomes a common thing that people talk about. That is when you can come with the modified mosquitoes. It is like that. If you do not do this then it may bring very big problem, and people may even attack you, chase you or embarrass you”* (Female).

FGD participants advised that, in order to win the trust of people, researchers would need to come up with means to show people the attributes of this technology, rather than just tell them. Village leaders explained that more efforts are still needed to educate people on different mosquito species, and on how to differentiate between malaria-transmitting and other mosquitoes. Without a degree of familiarity with these issues, it was noted that it would be impossible to convince people that the mosquitoes being released were harmless.

*“When you go there with your mosquitoes and tell them that you want to release them, they will ask you if the mosquitoes can harm them, and you will say that these are harmless mosquitoes. They will then ask you to prove it. How will you do that? You will have to find a way of demonstrating to people that these mosquitoes are harmless. If you just tell people that any mosquitoes are harmless you are in for trouble. We all know that all mosquitoes spread diseases, and that all mosquitoes are bad.”* (Male).

## Discussion

Historically, the release of modified mosquitoes has received a mixed response from the communities hosting these interventions [49, 50]. Current field research projects on mosquito modification include extensive campaigns of public information and engagement [30, 51, 52]. It has become abundantly clear that these campaigns must start well in advance of the deployment of the technology, and that they should be preceded by research into the concerns, expectations and interpretive frames that local residents bring to bear on the prospect of making disease control reliant on the introduction of altered mosquitoes into the environment [13, 27, 53].

This study attempted to explore perceptions of mosquito modification technologies in a region of southern Tanzania where no trials of modified mosquitoes have yet taken place, but where the epidemiology of malaria might in the near future recommend their use. This is a region, furthermore, where many other malaria control interventions have been piloted in the past, and where a significant proportion of the population is familiar with entomological research, thanks to the long-term presence of the Ifakara Health Institute [5]. This study provides the first social scientific evidence on public perspectives on mosquito modification in Tanzania.

Nearly all community members that responded to the survey reported no knowledge or prior awareness of mosquito modification technologies for malaria control. This is understandable, since no releases have taken place in the country to date, and local and national media have offered very limited coverage of debates on this issue elsewhere in the region. Similar findings have been observed in Mali and Nigeria [32, 54], for example, as well as in high-income countries such as the USA, where a 2016 survey indicated that 46% of respondents reported no prior information about gene-edited mosquitoes [55]. The generalized lack of knowledge and awareness made it difficult to assess in detail public perceptions of the technology, at least through a standardized survey questionnaire. FGDs were introduced to allow us to explore mosquito modification technologies in some detail with a select group of local residents, so as to study in depth the specific conceptual frames that might be used to make sense of the technology.

Although all FGD participants had never before heard about mosquito modification, they all expressed a great deal of fascination over this approach to malaria control once the discussions got underway. FGD participants associated the technology to several aspects of their lived experiences, specifically the practice of cross-breeding domestic animals to select for preferred traits, or the adoption of hybrid crop seeds that provide better yield and drought protection. The prospect that similar techniques could be used to eliminate malaria appeared, therefore, intuitively plausible, even before the specific principles of each form of mosquito modification were discussed.

The analogy with forms of biological modification familiar to local residents also shaped their initial consideration of risk, as it allowed them to balance any potential hazards the technology might carry with the promise of a direct benefit. Similar findings have been reported in the US, where support for genetic modification increased once the potential risks and benefits of the technology were communicated to the people [56]. A study by Widmar et al., for example, indicated that genetic modification was most acceptable when used in human medicine and in disease control [57]. In this case, participants were relatively supportive of the approach once mosquito modification was contrasted with other malaria control interventions, partly because it was seen as requiring less direct participation from the community, and because it was thought to reduce environmental risks they associated with other interventions (i.e. extensive use of chemicals in IRS, ITNs, or larviciding).

After being presented with several forms of modification, participants expressed the greatest interest in gene drive applications, particularly male-biased sex-distorting alterations. This was due to the low perception of risk associated with male mosquitoes and the high perception of risk associated with female mosquitoes. Previous research in the study site indicate near universal awareness in the community that malaria is transmitted by female *Anopheles* mosquitoes, and that male mosquitoes do not transmit any diseases [38, 58]. The participants also pointed out that the gene drive approach would require fewer and smaller releases compared to other mosquito modification technologies [15, 18].

FGD participants contemplated the possibility that modified mosquitoes would look or behave differently than local mosquitoes, and sought further clarification on this particular point. These concerns, although expressed mildly in this case, have led to major controversies over the release of modified mosquitoes in the past. Examples include fears that mutations in the mosquito itself, or in the pathogen, could result in higher rates of disease transmission in the future, or that the modification introduced in the mosquito could be transmitted to humans through biting [32, 33, 59]. It is crucial that these concerns are given careful consideration, and that researchers and sponsors of these technologies are in a position to allay these fears with adequate scientific evidence.

Participants in our FGDs also expressed the concern that eliminating just one mosquito species would not be enough, and would fail to garner sufficient public support for the intervention. This concern can be explained by the fact that people are generally unable to differentiate between malaria vectors and other mosquito species, and that the effectiveness of most other malaria vector control interventions is assessed against a reduction of overall mosquito density. It is estimated that malaria vectors in this region account for less than 10% of the overall mosquito population [5, 60], and some key vector species, such as *Anopheles funestus*, represent a small proportion of anophelines. A technology targeting only a key vector species might be seen as not working if the community experiences little difference in their overall exposure to mosquito nuisance.

Addressing these perceptions and concerns will require a proactive strategy of public outreach. Community engagement in public health research needs to go beyond simply providing the community information or consulting users for their views. An effective program demands building durable partnerships between researchers and the community, eliciting and addressing concerns in terms that resonate locally, and through a process that is embedded within, rather than abstracted from, their everyday lives [27].

Participants in our study emphasized that it would not be enough to simply raise awareness about these technologies; people needed to be fully engaged in order to make sense of the technology in their specific context. They stressed the need to demonstrate, rather than tell, the safety and effectiveness of the intervention. Similar findings have been observed in studies carried out in Mali and Nigeria, where respondents asked that evidence of the technology’s safety and effectiveness be provided before they could allow it in their settings [32, 54]. These discussions suggest that education is an iterative process, and that the provision of the facts of how the technology works is only a first step. To truly grasp the public health potential and significance of mosquito modification, communities would need to be able to contextualise these technologies within their everyday life, to translate abstract technical operations into practical concerns.

This study is not without limitations. Only two FGD sessions were conducted, which is a rather small sample size, and the community leaders that participated in the discussions represent a particular segment of the population. Additionally, the study was conducted among communities that have long been associated with public health and entomological research campaigns through Ifakara Health Institute and, therefore, are knowledgeable about malaria transmission and prevention. These limitations to generalizability notwithstanding, the two groups still generated a wealth of qualitative data on the preferred interpretive frames and the most salient concerns that local residents in a rural, malaria-endemic region of Tanzania express in relation to the prospect of using modified mosquitoes as a public health tool. Further studies should be undertaken in communities that may be less familiar with malaria control practices, and to explore in greater depth responses to specific forms of mosquito modification. This study can serve as a baseline from which to develop more granular investigations of local concerns and perceptions, and upon which to build a robust and effective set of tools for public engagement.

## Conclusions

Understanding how communities perceive and interpret new public health technologies is crucial in generating durable support for these interventions. This study offers vital clues on how rural communities without prior awareness of mosquito modification technologies respond to the prospect of using genetically-modified mosquitoes as a tool for malaria control. Despite the lack of prior knowledge, FGD participants offered a set of distinctive interpretive frames to interpret mosquito modification technologies, referring in particular to their experiences selecting preferred traits in domestic plants and animals through cross-breeding. These interpretive frames and locally resonant analogies provide a basis for effective community engagement to address any specific concerns, support further social scientific research, and potentially aid in the future development and deployment of such technologies for malaria elimination. The findings of this study may find broader application in other settings where GMMs or similar approaches are being planned.

## Data Availability

All data for this study will be available upon request.
