# Correction to: Hybrid mosquitoes? Evidence from rural Tanzania on how local communities conceptualize and respond to modified mosquitoes as a tool for malaria control

**DOI:** 10.1186/s12936-021-03777-0

**Published:** 2021-06-09

**Authors:** Marceline F. Finda, Fredros O. Okumu, Elihaika Minja, Rukiyah Njalambaha, Winfrida Mponzi, Brian B. Tarimo, Prosper Chaki, Javier Lezaun, Ann H. Kelly, Nicola Christofdes

**Affiliations:** 1 Environmental Health and Ecological Science Department, Ifakara Health Institute, P. O. Box 53, Ifakara, Tanzania; 2 School of Public Health, Faculty of Health Sciences, University of the Witwatersrand, 1 Smuts Avenue, Braamofontein, 2000 South Africa; 3 Institute of Biodiversity, Animal Health and Comparative Medicine, University of Glasgow, Glasgow, G12 8QQ UK; 4 School of Life Science and Bioengineering, Institution of Science and Technology, The Nelson Mandela African, P. O. Box 447, Arusha, Tanzania; 5 Institute for Science, Innovation and Society, School of Anthropology and Museum Ethnography, University of Oxford, Oxford, UK; 6 Department of Global Health and Social Medicine, King’s College London, London, UK

## Correction to: Malar J (2021) 20:134 https://doi.org/10.1186/s12936-021-03663-9

Following publication of the original article [1], it was brought to our attention that the article had published with an incorrect Funding declaration.

The declaration has been corrected in the published article and may be seen below:

- “This work was supported by the Consortium for Advanced Research Training in Africa (CARTA), awarded to MFF. CARTA is jointly led by the African Population and Health Research Center and the University of the Witwatersrand and funded by the Carnegie Corporation of New York (Grant No—G-19-57145), Sida (Grant No: 54100113), Uppsala Monitoring Centre and the DELTAS Africa Initiative (Grant No: 107768/Z/15/Z). The DELTAS Africa Initiative is an independent funding scheme of the African Academy of Sciences (AAS)’s Alliance for Accelerating Excellence in Science in Africa (AESA) and supported by the New Partnership for Africa’s Development Planning and Coordinating Agency (NEPAD Agency) with funding from the Wellcome Trust (UK) and the UK government. The statements made and views expressed are solely the responsibility of the Fellow. This work was also supported by the Bill and Melinda Gates Foundation (Grant Number: OPP1177156), Howard Hughes Medical Institute (Grant Number: OPP1099295) and by Application of Novel Transgenic technology & Inherited Symbionts to Vector Control (ANTI-VeC) (Grant Number: AVPP0027/1), all awarded to Ifakara Health Institute.”

The authors apologize for any inconvenience caused.
